# Implementing leading practices in regional-level primary care workforce planning: Lessons learned in Toronto

**DOI:** 10.1177/08404704221117263

**Published:** 2022-10-14

**Authors:** Sarah Simkin, Caroline Chamberland-Rowe, Cynthia Damba, Nathalie Sava, Ting Lim, Ivy Lynn Bourgeault

**Affiliations:** 1 12365 University of Ottawa, Ottawa, Ontario, Canada.; 2 56004 University of Ottawa, Ottawa, Ontario, Canada.; 3 573450 Ontario Health, Toronto, Ontario, Canada.; 4 508783 Unity Health Toronto, Toronto, Ontario, Canada.; 5 151181 University of Ottawa, Ottawa, Ontario, Canada.

## Abstract

Investment in capacity for implementation of leading practices in regional-level health workforce planning is essential to support equitable distribution of resources and deployment of a health workforce that can meet local needs. Ontario Health Toronto and the Canadian Health Workforce Network (CHWN) co-developed and operationalized an integrated workforce planning process to support evidence-based primary care workforce decision-making for the Toronto region. The resultant planning toolkit incorporates planning processes centred around engagement with stakeholders, including environmental scanning tools and a quantitative planning model. The outputs of the planning process include estimates of population need and workforce capacity and address challenges specific to Toronto, such as patient mobility, anticipated rapid population growth, and physician retirement. We highlight important challenges and key considerations in the development and operationalization of workforce planning processes, particularly at the regional level.

## The planning imperative

Ontario Health Toronto (Toronto Region)^1^ plans, funds, implements, and evaluates local health services and is tasked with optimizing access to integrated services across the Toronto Region. To respond to this mandate, and to support evidence-based decision-making regarding the deployment of the workforce and other health system resources, a robust approach to primary care workforce planning is needed.

The planning landscape in Toronto is complex given the cultural and socio-economic diversity of the population, population mobility, and variations in health service use, health outcomes, and distribution of service providers across the 140 neighbourhoods in the city. A tailored workforce planning approach that addresses the core needs and key challenges present within the Toronto Region’s primary care landscape is, therefore, required. The planning toolkit needs to: (1) enable population needs-based and evidence-informed planning; (2) be integrated and multi-professional, including all the providers and services involved in the delivery of primary care; (3) facilitate an assessment of alignment between population needs and workforce capacity at the neighbourhood, sub-region, and whole city levels; (4) accommodate short planning horizons, in line with regional decision-making timelines; and (5) address certain challenges specific to Toronto, such as patient mobility, population growth, and anticipated physician retirement.

## Process

In 2017, the Health Analytics team at Ontario Health Toronto partnered with experts from the Canadian Health Workforce Network to co-develop and operationalize a fit-for-purpose toolkit to support integrated primary care workforce planning in the Toronto Region. For this project, which has spanned two phases of work to date, we adopted an approach informed by a participatory action research framework^2^ involving continuous collaboration between Ontario Health Toronto and the Canadian Health Workforce Network, and extensive consultations with community, health system, and data stakeholders. This approach builds capacity for and commitment to primary care workforce planning within the Toronto Region, while simultaneously enhancing the acceptability and validity of the toolkit and its outputs.

### Phase 1: 2017-2018

In Phase 1, we conducted a targeted review and assessment of existing planning models.^3^ Models were assessed for suitability based on guiding principles related to the unique planning needs of the Toronto Region (identified *a priori*). Since no single model fully met these needs, the final approach combined key features from existing models (including England’s Robust Workforce Planning Framework,^4^ Australia’s Health Workforce Planning Tool^5^ and several tools from New Zealand’s Workforce Intelligence and Planning Framework).^6^

**Figure 1.**
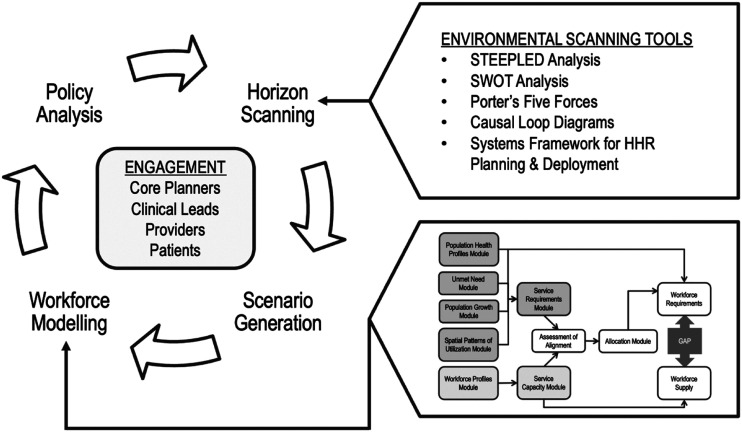
Overview of primary care workforce planning toolkit.

The final health workforce planning toolkit,^7^ summarized in Figure 1, includes a cyclical process for primary care workforce planning, as well as a fit-for-purpose quantitative workforce planning model. The development of the quantitative model was informed by an environmental scan and assessment of available data sources.^8^ Data related to population health need and profession-specific health workforce supply were identified and assessed for quality, availability and comprehensiveness, as well as overall suitability for use in modelling. Given Toronto’s cultural diversity, variables that could help apply an equity lens to workforce planning, such as gender, language, race, Indigenous identity, and disability were specifically sought and considered.

Inspired by the process prescribed by the UK’s Centre for Workforce Intelligence,^9^ the toolkit’s cyclical workforce planning process includes horizon scanning (systematic exploration of the likely future developments, driving forces, and potential issues that could influence workforce requirements), scenario generation (development and elaboration of a range of plausible futures), and a quantitative workforce planning model, all of which are intended to inform policy analysis and decision-making. Environmental scanning tools and the modular quantitative model (described in Textbox 1) enable planners to engage with primary care providers, community stakeholders, and Ontario Health Toronto’s network of partners on an ongoing basis. Both planning and engagement are iterative and dynamic processes that allow planning to respond to emerging issues and challenges.

Textbox 1: Modules of the quantitative workforce planning model

- The **Population Health Profiles Module** captures characteristics of the population that impact the need for primary care services.
- The **Population Growth Module** captures neighbourhood-level population growth projections generated by the City of Toronto, allowing us to adjust service requirements to account for anticipated population growth.
- The **Spatial Patterns of Utilization Module** captures a snapshot of primary care utilization patterns and allows us to adjust service requirements to account for patients’ care-seeking behaviours.
- The **Service Requirements Module** estimates primary care service requirements using the CIHI Population Grouping Methodology.
- The **Workforce Profiles Module** captures information about the primary care workforce—including physicians and allied health providers—practicing in each neighbourhood.
- The **Service Capacity Module** estimates the capacity of the workforce to provide primary care services.

### Phase 2: 2019-2022

**Figure 2.**
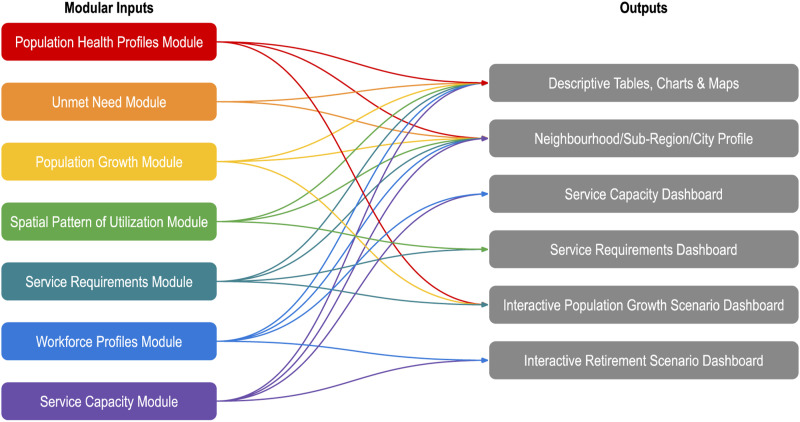
Data flow between modules and outputs.

In Phase 2, we operationalized primary care workforce planning using the toolkit developed in Phase 1. We conducted a full-day facilitated consultation session with stakeholders at the outset of this phase of work that included horizon scanning exercises and resulted in a Statement of Purpose, which served to define the goal and scope of the first cycle of workforce planning: “To build a body of evidence around the current (and projected future) states of population health needs and primary care service provision at a neighbourhood level within Toronto.” We also garnered input from stakeholders as to priority scenarios for the region (population growth and physician retirement), which in turn guided the development of the project outputs. We accessed data from multiple sources to populate the quantitative model (Table 1). The model has a number of modules, which take the form of automated spreadsheets that capture all relevant data and produce intelligence about the population and the workforce. Data from these modules are used to generate descriptive tables, charts, and maps, as well as static and interactive dashboards, to synthesize and summarize emerging findings (Figure 2). In this phase of work, we also conducted outreach exercises with local primary care leaders to validate the results and optimize the utility of the outputs.

**Table 1.** Sources of data mobilized for the quantitative workforce planning model.

Population Health Profiles and Unmet Need Modules	Ontario Community Health Profiles Partnership (OCHPP)
Population Growth Module	Neighbourhood-level population growth estimates from the City of Toronto Planning Department
Spatial Patterns of Utilization Module	Utilization Matrix generated using data from ICES through an Applied Health Research Question (AHRQ) request
Service Requirements Module	CIHI population grouping methodology outputs provided by the Ontario Ministry of Health
Workforce Profile and Service Capacity Modules (physicians)	ICES Physician Database (IPDB) accessed through OCHPP
Workforce Profile and Service Capacity Modules (other health professionals)	Health Professions Database (HPDB) outputs provided by the Ontario Ministry of Health

### Phase 3

The third phase of work, which initiated in September 2022, will involve updating the data in all the modules, building capacity for primary care workforce planning within and beyond the Toronto Region, developing and implementing additional modules that respond to the planning needs of local stakeholders (Textbox 2), and evaluating the impact of the toolkit.

Textbox 2: Modules targeted for operationalization in Phase 3

- The **Unmet Need Module** captures information enabling the quantification and remediation of neighbourhood-level unmet healthcare need, which can contribute to an adjustment of service requirements.
- After an initial assessment of alignment between population service requirements and workforce service capacity, an optional **Allocation Module** allows services to be shifted between primary care providers with overlapping scopes of practice, or to virtual care, to optimize alignment and minimize the gap between service requirements and service capacity.
- After consideration of Unmet Need and various Allocation scenarios, gaps in alignment between population need and workforce service capacity may remain. The **Education/Training Module** will explore how these gaps can be filled, taking into account the context, needs, and resources of the local community as well as interprofessional comprehensive care competencies, retraining of existing professionals, and integration of internationally educated professionals into the workforce.

## Key outputs

Through the first cycle of workforce planning, we produced a variety of outputs designed to support evidence-informed decision-making at the neighbourhood, sub-region, and city levels.

A neighbourhood package available for each neighbourhood across Toronto includes a series of three static dashboards that bring together information about the population, the workforce, and the alignment of the two. Sub-region packages, which collate the static dashboard outputs for all neighbourhoods in a given sub-region, are also available for the ease of reference of users. Finally, a city package is available, including a static city-level profile and a series of descriptive maps. Interactive dashboards and more detailed information about specific communities are available upon request through Ontario Health Toronto.

In line with our commitment to ensuring that the toolkit, and its outputs are open-access and readily available for use by health system stakeholders within and beyond the Toronto Region, the tools we have developed are available on the Canadian Health Workforce Network website^10^ and all of our static outputs are posted on the Ontario Community Health Profiles Partnership (OCHPP) web site.^11^

Our integrated knowledge translation^12^ activities have also involved a series of tailored presentations to health system stakeholders who contributed to toolkit development and operationalization, or who are interested in making use of the toolkit and/or, its outputs. Ontario Health Toronto will support ongoing engagement with interested users and provide assistance in the development and interpretation of targeted scenarios.

## Impact

The outputs from this integrated primary care health workforce planning approach benefit Toronto region providers, planners, and stakeholders broadly. The toolkit helps characterize primary care patients (where they come from and their primary care needs) and uses leading practices (such as the CIHI Population Grouping Methodology^13^) to generate an estimate of the primary care resources needed in communities across Toronto. Adjustment for patient mobility and consideration of future population growth respond to planning challenges that are specific to Toronto and further enhance the utility of the outputs.

Decision-makers in Toronto and elsewhere are in urgent need of support to identify high-needs areas and future emerging primary care needs, taking into account population growth, demographic shifts, provider retirement, and changing practice patterns. Our outputs respond to a need for evidence to support decisions such as where new physician positions would best be placed, which community would benefit most from new or expanded integrated primary care teams, where best to allocate additional funding, and where other allied health resources should be deployed. Planning processes such as ours provide this evidence, along with the foundation and scaffolding to support the development of essential local and regional primary care planning capacity.

## Challenges and limitations

Neighbourhood-level planning is driven by the aspiration to provide comprehensive primary care close to home for all patients. And yet, mobility of patients and service providers continues to be a challenge for decision-makers. Incorporating adjustments for spatial patterns of utilization has helped to explain observed utilization patterns and made neighbourhood-level planning meaningful. But care-seeking patterns are dynamic, and they influence—and are influenced by—the primary care landscape, so the importance of iterative planning with regular timely data and methodological updates cannot be overstated.

Health workforce data are critical, not only to inform deployment of resources, but also to assist with evaluation of workforce-related health system interventions. And yet, availability and accessibility of workforce data to regional-level planners is a major barrier to evidence-informed decision-making. Record-level data for physicians and the allied health professions that are high quality, granular, comprehensive, and timely are often difficult to access. Our team invested significant time and effort to identify high quality datasets that captured the data elements required to populate our quantitative model, and to design robust and reliable methodologies and automated analyses that could be run by third parties and output in aggregate at the neighbourhood-level. Delays in access to data significantly extended the timelines for the project, and data lags and limited longitudinal information constrain extrapolation, and in some cases undermine the accuracy of our projections. Engagement with local stakeholders will help us to estimate the degree of uncertainty that is associated with our results.

We have had to adapt to an evolving landscape of health system transformation, although most of the workforce planning issues facing decision-makers in Toronto have remained pertinent or become more urgent. Regular engagement with stakeholders and decision-makers confirmed that the planning priorities and project outputs remain relevant. The iterative nature of the planning process and the modular model allowed us to easily consider the impact of additional scenarios (such as early retirements) and we are confident that future emerging issues (such as virtual care and the changing roles of pharmacies) can be addressed in a similar manner.

## Key success factors

Throughout the project, we committed to adopting and championing leading practices in planning (identified in Phase 1), including the use of high quality and comprehensive data. The Ontario Health Professions Database^14^ (HPDB), a rich dataset that brings together standardized, consistent, and comparable demographic, geographic, educational, and employment information on all regulated health professionals in Ontario, was an important facilitator of integrated planning for this project. Likewise, the Canadian Institute for Health Information Population Grouping Methodology^8^ was an enabler of regional-level needs-based planning.

Consultation and reciprocal engagement with stakeholders helps to build trust in the planning process. Throughout the project, we consulted widely with our partners (frontline providers, primary care leadership, the City of Toronto, and numerous data stewards and subject matter experts) to define planning priorities and to develop and refine the planning methodology. For example, at the outset of the project, we engaged in broad consultations before delineating the professions and professional activities that can play a part in comprehensive primary care (Table 2). We have developed tailored presentations, workshops, and educational materials (such as User Guides) to help stakeholders understand and use the outputs, further building capacity for planning both within the core planning team and across distributed primary care leadership structures. A framework that prioritizes consultation and engagement is essential to ensure that local barriers and challenges can be addressed, and to maintain the utility and credibility of the planning process.

**Table 2.** Professions and activities in our comprehensive definition of primary care.

Primary care professions	Primary care activities
• Chiropodists• Dieticians• Midwives• Nurse practitioners• Optometrists• Occupational therapists• Pharmacists• Psychologists• Physicians• Physiotherapists• Registered nurses• Registered practical nurses• Respiratory therapists• Speech-language pathologists	• General service provision• Continuing care• Comprehensive primary care• Chronic disease prevention and management• Public health• Mental health and addiction• Primary maternity care• Geriatric care• Infectious disease prevention and control• Palliative care

## What we have learned and want to share

This project is a case study in leading practice health workforce planning. It responds to an urgent need for intelligence to support evidence-informed health system decision-making in Toronto. The process emphasizes collaboration with stakeholders and incorporation of local knowledge to supplement the toolkit outputs, validate the results, and build a fulsome picture of local primary care needs. Having a holistic view of the various factors at play can help local decision makers to exercise flexibility and ingenuity in developing innovative solutions to address primary care service gaps.

We have built a strong foundation for comprehensive primary care workforce planning. The toolkit and model will be shared with others doing primary care workforce planning to spread and scale this type of leading practice and to promote use of a common methodology. But more needs to be done: in keeping with the iterative nature of planning, the model will be refined on an ongoing basis, to incorporate updated population data and more current primary care utilization patterns and workforce information. Over time, through continuous cycles of iterative methodological refinement, the accuracy of estimates produced by the model will be evaluated and enhanced.

At the policy level, our experience indicates that there is a need for decision-makers to recognize the importance of planning and support the development of planning capacity, literacy, and engagement. Targeted investments in data and data infrastructure, tools to support planning, and supported networks of leaders, clinicians, analysts, and other stakeholders committed to the principles and promise of health workforce planning will be essential to the success of future planning endeavours. We encourage aspiring planners to adopt leading practices, to anticipate challenges and advocate for their resolution, and to prioritize engagement and collaboration in their health workforce planning endeavours.
